# Testing olfactory dysfunction in acute and recovered COVID-19 patients: a single center study in Italy

**DOI:** 10.1007/s10072-021-05200-7

**Published:** 2021-03-26

**Authors:** Jacopo Pasquini, Carlo Maremmani, Stefano Salvadori, Vincenzo Silani, Nicola Ticozzi

**Affiliations:** 1 Department of Neurology and Stroke Unit and Laboratory of Neuroscience, Istituto Auxologico Italiano IRCCS, Milan, Italy; 2 Department of Pathophysiology and Transplantation, Dino Ferrari Center, Università degli Studi di Milano, Milan, Italy; 3 Neurology Unit, Ospedale Apuane - Azienda USL Toscana-Nord Ovest, Massa, Italy; 4 Institute of Clinical Physiology, National Research Council (CNR), Pisa, Italy

**Keywords:** Anosmia, Hyposmia, Olfactory testing, COVID-19, SARS-CoV-2

## Abstract

**Background:**

Olfactory dysfunction in coronavirus disease 2019 (COVID-19) is common during acute illness and appears to last longer than other symptoms. The aim of this study was to objectively investigate olfactory dysfunction in two cohorts of patients at two different stages: during acute illness and after a median recovery of 4 months.

**Methods:**

Twenty-five acutely ill patients and 26 recovered subjects were investigated. Acute patients had a molecular diagnosis of COVID-19; recovered subjects had a positive antibody assay and a negative molecular test. A 33-item psychophysical olfactory identification test tailored for the Italian population was performed.

**Results:**

Median time from symptoms onset to olfactory test was 33 days in acute patients and 122 days in recovered subjects. The former scored a significantly higher number of errors at psychophysical testing (median [IQR]: 8 [13] vs 3 [2], *p* < 0.001) and were more frequently hyposmic (64% vs 19%, *p* = 0.002). Recovered subjects reported a variable time to subjective olfactory recovery, from days up to 4 months. Participants included in the study reported no significant nasal symptoms at olfactory testing. Among recovered subject who reported olfactory loss during acute COVID-19, four (27%) were still hyposmic. Demographic and clinical characteristics did not show significant associations with olfactory dysfunction.

**Conclusion:**

Moderate-to-severe hospitalized patients showed a high level and frequency of olfactory dysfunction compared to recovered subjects. In the latter group, subjects who reported persisting olfactory dysfunction showed abnormal scores on psychophysical testing, indicating that, at least in some subjects, persistent hyposmia may represent a long-term sequela of COVID-19.

**Supplementary Information:**

The online version contains supplementary material available at 10.1007/s10072-021-05200-7.

## Introduction

Olfactory dysfunction in coronavirus disease 2019 (COVID-19) is a common symptom appearing during the acute phase of the disease [1–5]. The frequency and degree of olfactory dysfunction during COVID-19 was assessed in some studies through heterogeneous psychophysical olfactory tests, mostly in mild disease [6–10]. These reports have shown high prevalence of olfactory dysfunction, from 40 to 98%. One study in hospitalized patients found that about 40 days after symptoms onset, olfactory dysfunction could be detected in 21% of patients [11]. Conversely, little is known about subjective and objective recovery of olfactory function in COVID-19, as only a few studies have been performed and their observation period was limited from 2 to 8 weeks after symptoms onset [12–15]. While these studies have shown relatively high recovery rates in the first weeks after symptoms onset, they still report around 40% of patients affected by olfactory dysfunction at the end of their follow-up. Overall, olfactory dysfunction in hospitalized patients with moderate-to-severe disease and long-term recovery of olfactory function after COVID-19 have not been well established.

In this study we aimed to describe the features of COVID-19-associated olfactory dysfunction in hospitalized patients with acute, moderate-severe COVID-19 as well as in recovered subjects whose symptoms onset was at least 3 months prior. Olfactory dysfunction was investigated through psychophysical testing that was carried out with a 33-item suprathreshold olfactory test for which normative values have previously been determined in a population of 511 healthy controls [16].

## Methods

### Study design

This cross-sectional, two-phase study was designed to evaluate and compare COVID-19 olfactory dysfunction in acutely ill patients and recovered subjects. A cohort of 25 COVID-19 moderate-severe patients was enrolled while hospitalized at our institution in April 2020; a second cohort of 26 COVID-19 recovered subjects was enrolled between June and September 2020.

### Participants’ selection

The study was conducted at Istituto Auxologico Italiano IRCCS, Milan, Italy. Exclusion criteria for all participants were pre-existing olfactory or taste disturbances; invasive and non-invasive mechanical ventilation; oxygen therapy and inability to tolerate room air for less than 30 min; ongoing acute or chronic sinusitis; past severe head trauma; Parkinson disease, Alzheimer disease or other types of dementia; and inability to communicate verbally with the clinician (e.g., language barrier).

The cohort of 25 acute COVID-19 patients was selected among 54 patients who were present at our institution between 20th April and 30th April 2020. All patients had a diagnosis of COVID-19 by SARS-CoV-2 RNA nucleic acid amplification test (NAAT) on naso-oropharyngeal swab.

Twenty-six recovered subjects were selected among employees of Istituto Auxologico Italiano and close relatives who a positive COVID-19 antibody assay that was carried out as a screening program. All tested negative at a subsequent SARS-CoV-2 RNA NAAT. Eight of these subjects had a molecular diagnosis through NAAT during acute disease. Serological testing was carried out with either one of two commercially available kits (Diasorin Liaison® SARS-COV-2 S1/S2, Roche Elecsys® Anti-SARS-CoV-2).

Before olfactory testing, all participants underwent a structured interview regarding olfactory or gustatory disturbances before and during the disease, and the total nasal symptom score questionnaire (3 items, total score 0–9) [17] (Supplementary Material).

### Psychophysical olfactory testing

All participants were tested without respiratory personal protective equipment (e.g., surgical mask). Psychophysical olfactory testing was performed with the Italian Olfactory Identification Test (IOIT), which includes 33 odorants, each one absorbed into a small, rectangular, white cardboard testing card contained inside a sealed aluminum envelope. All components of the test, i.e., 33 small aluminum envelopes each containing a testing card, are for single-use and disposable. Each envelope is numbered and matched with the corresponding multiple-choice question on the answer sheet; each multiple-choice question is composed of four possibilities, and only one is the correct answer. Once an aluminum envelope is opened, the odor is released and the patient is asked “Do you smell something? If so, what smell do you detect?.” The patient has to pick an answer even in the case no odor is detected. The 33 odorants are commonly present in the Italian culture and cover a wide range: citrus, floral, sweet, wood-like, minty, and unpleasant (full list in Supplementary Material). The upper reference limits of errors, above which the test is considered abnormal and the subject hyposmic, are derived from an Italian cohort of 511 healthy volunteers. Normative values have been previously computed using mean + 2 standard deviations; the 97.5 percentile; and 95% individual confidence curves of a third-order polynomial regression. They are the same for males and females: 4 errors below age 49, 5 errors between 50 and 59, 6 errors between 60 and 69, 7 errors over 70 years of age [16].

### Statistical analysis

Descriptive statistics are reported as numbers and percentages, or medians and interquartile ranges (IQRs). Comparisons between two groups were performed with non-parametric tests. In order to compare the central values of a quantitative variable (e.g., number of errors at psychophysical testing) between two groups, Mann-Whitney *U*-test was performed. The comparison of the distribution of a continuous variable (e.g., age) across two groups was performed with the Kolmogorov-Smirnov two-sample test. In order to test the relationship between two categorical variables (e.g., sex across two groups) the Fisher’s exact test was used, while for two continuous or ordinal variables Spearman rank test was used. The significance threshold for hypothesis testing was *p* < 0.05. Statistical analysis was carried out with Statistical Package for Social Sciences (IBM® SPSS) version 21.

## Results

Fifty-one subjects were included in the study: 25 acute COVID-19 hospitalized patients and 26 recovered subjects. Median (IQR) age was 52.1 (24.1) years and 27 subjects (53%) were female. Recovered subjects were significantly younger than hospitalized patients (*U* = 59.0, *p* < 0.001). Sex across the two groups was evenly distributed (χ^2^ = 3.296, *p* = 0.095). Demographic and general clinical characteristic are shown in Table 1.

**Table 1:** Demographic and general clinical characteristics of the 51 participants enrolled in the study

	Hospitalized patients	Recovered subjects
Number	25	26
Sex, M/F	15/10	9/17
Age, median (IQR)	66.7 (19.1)	46.4 (12.5)
Asymptomatic/symptomatic for COVID-19^1^, *n*	0/25	5/21
Interstitial pneumonia at chest imaging (X-ray or CT scan), *n*	23 (92%)	2 (8%)

^1^Symptoms included: fever, cough, dyspnea, diarrhea, nasal congestion, rhinorrhea, olfactory and taste disturbances

*COVID-19*, coronavirus disease 2019; *CT*, computed tomography

No subject included in the study reported olfactory loss as the first isolated symptom. Four patients in each cohort reported the onset of olfactory symptoms to be concomitant with other COVID-19 symptoms.

Median time (IQR) from first symptoms to psychophysical olfactory testing was 33 (9) days for hospitalized patients and 122 (51) days for recovered subjects; the latter group was tested at least 90 days after symptoms onset. At the time of testing, no participant showed rhinorrhea, nasal obstruction, or other nasal symptoms; all had a TNSS of zero or one.

The number of errors at psychophysical olfactory testing was significantly higher in the hospitalized group compared to the recovered group (median [IQR]: 8 [13] vs 3 [2], *U* = 114.0, *p* < 0.001). Based on psychophysical olfactory test normative values, the proportion of hyposmic subjects was significantly higher in hospitalized patients compared to recovered subjects (64% and 19%, respectively, χ^2^ = 10.546, *p* = 0.002; Table 2).

**Table 2:** Summary of characteristic of olfactory disturbances in the two groups included in the study

	Hospitalized patients (*n* = 25)	Recovered subjects (*n* = 26)	*p*
Subjective olfactory or gustatory disturbances during COVID-19, *n* (%)	10 (40%)	15 (57%)	–
Subjective olfactory or gustatory disturbances at olfactory test administration, *n* (%)	3 (12%)	4 (15%)	–
Time from symptoms onset to olfactory testing, median days (IQR), range	33 (12), 9–51	122 (51), 91–171	–
Number of errors at psychophysical olfactory test, median (IQR), range	8 (13), 1–24	3 (2), 0–13	<0.001^1^
Hyposmia at psychophysical olfactory test, *n* (%)	16 (64%)	5 (19%)	0.002^2^
Hyposmic subjects, M/F, number (percentages^3^)	12/4 (80%/40%)	2/3 (22%/18%)	

^1^Mann-Whitney *U*-test

^2^Fisher’s exact test

^3^Percentages are based on the number of subjects of the same sex in each group

### Olfactory and clinical characteristics of hospitalized patients

Eight patients (32%; four females) experienced subjective olfactory disturbances during the disease, and of these, seven noted also gustatory disturbances. Three (12%) of those eight patients reported persisting subjective olfactory disturbances at the time of psychophysical testing, while five declared a subjective recovery of olfactory function. Interestingly, abnormal IOIT scores were observed in all patients who complained of subjective hyposmia at the time of testing, but also in thirteen individuals (52%) unaware of their deficit. These results are summarized in Table 3. Overall, four of ten females and twelve of 15 males (total: 16 of 25, 64%) showed olfactory dysfunction (χ^2^ = 4.167, *p*-value = 0.087). Age distribution in hyposmic and normosmic patients did not differ significantly (median age [1st–3rd quartiles] 68 [59–77] and 58 [49–67]; *d* = 0.883, *p*-value = 0.416).

**Table 3:** Distribution of hospitalized patients based on subjective perception of olfactory function and olfactory test result at the time of olfactory testing

	Subjective normosmia, *n* (%)	Subjective hyposmia, *n* (%)	Total, *n* (%)
Olfactory test result: normosmia, *n* (%)	9 (36%)	0 (0%)	9 (36%)
Olfactory test result: hyposmia, *n* (%)	13 (52%)	3 (12%)	16 (64%)

*n*, number of patients

Eighteen patients (72%) needed some kind of respiratory support during the disease (all oxygen therapy; twelve non-invasive mechanical ventilation and of these, three needed invasive mechanical ventilation; all were tested once they could tolerate room air for at least 30 min). The presence of olfactory dysfunction and the number of errors at olfactory testing were not associated with age, disease duration, and the need for mechanical ventilation (Supplementary Table 1). Two patients died due to in-hospital complications (one pneumomediastinum, one multi-organ failure). The remaining patients were discharged from the COVID unit. These 23 patients underwent a follow-up telephone interview in February 2021, in order to assess their subjective level of smell: five previously normosmic patients did not report any smell complaint; three patients with COVID-19 hyposmia reported a partial recovery; six patients with either subjective smell loss or abnormal olfactory test score reported a complete recovery; and nine patients were lost to follow-up (one died, eight could not be reached by telephone).

### Olfactory and clinical characteristics of recovered subjects

All subjects in the recovered group had evidence of prior COVID-19 infection detected through antibody testing and a concomitant negative SARS-CoV-2 RNA NAAT on nasopharyngeal swab. Five subjects did not have a history of COVID-19 symptoms. Eight subjects had a molecular diagnosis of COVID-19 during the acute phase through NAAT.

Fifteen subjects (58%) reported subjective olfactory disturbances (anosmia or severe hyposmia) during acute COVID-19; eleven of these also complained gustatory problems. Two patients reported only gustatory disturbances. At the time of olfactory testing, eleven of 15 subjects reported a complete recovery. At psychophysical testing, a total of five subjects (19%, three females and two males) were hyposmic, while twenty subjects (77%, including all asymptomatic subjects) who described a subjectively normal olfactory function had normal test scores; one subject who complained persistent hyposmia following SARS-CoV-2 symptomatic infection had a normal test score (Table 4). Subjective median time to recovery in eleven subjects who reported full recovery of olfactory function was 45 days (IQR: 43; range: 9–100; Table 2).

**Table 4:** Distribution of recovered patients based on subjective perception of olfactory function and olfactory test result at the time of olfactory testing

	Subjective normosmia, *n* (%)	Subjective hyposmia, *n* (%)	Total, *n* (%)
Olfactory test result: normosmia, *n* (%)	20 (77%)	1 (4%)	21 (81%)
Olfactory test result: hyposmia, *n* (%)	2 (8%)	3 (11%)	5 (19%)

*n*, number of patients

No relationship was found between the number of errors at psychophysical testing and the number of days since COVID-19 symptoms onset (rho = −0.166, *p* = 0.472). The number of errors at olfactory testing was associated with age (rho = 0.530, *p* = 0.05) but the distribution of age in subjects with and without hyposmia was not significantly different (median age [1st–3rd quartiles] 49 [47–54] and 42 [36–48], respectively; *d* = 1.244, *p*-value = 0.091).

## Discussion

In this study we investigated COVID-19-related olfactory dysfunction through clinical interviews and psychophysical testing. We carried out a cross-sectional, comparative study in moderate-severe COVID-19 hospitalized patients and subjects who had recovered from prior SARS-CoV-2 infection. Psychophysical testing was performed with a 33 odor identification test previously validated in a population of 511 Italian healthy volunteers [16].

While recent evidence based on psychophysical testing has shown a high prevalence of olfactory dysfunction in the acute phase of the disease [6, 8, 10], little is known about long-term olfactory function recovery. Indeed, only a few studies assessed olfactory function using psychophysical tests, and all were limited to maximum 8 weeks after symptoms onset [12, 13, 15]. In our analysis, all recovered subjects were tested at least 90 days after symptoms onset and more than half after 4 months.

Awareness of olfactory dysfunction was low in the hospitalized group and high in the recovered group. In the latter, only three of 26 patients had conflicting results between subjective perception and psychophysical test. In hospitalized patients, low awareness of olfactory dysfunction may have been shadowed by the presence of more troubling symptoms, hospitalization, and respiratory support techniques. Data of the hospitalized group, in which 64% of patients were hyposmic, were collected during the early phase of the epidemic in Northern Italy and are in agreement with other studies carried out in Italy [10] and elsewhere [6–8]. Varying frequencies of olfactory dysfunction are likely related to psychophysical testing heterogeneity and different time intervals from symptoms onset to testing. We tested our patients a median of 33 days after symptoms onset: those who were tested later in the disease course needed non-invasive or invasive mechanical ventilation, thus could not be tested earlier.

A few recent prospective studies investigated the reversibility of olfactory dysfunction in COVID-19 [12, 13]. In a hospital-based study in Iran, patients were tested twice with the country’s version of the UPSIT at an interval of 1 or 4 weeks. They showed that 60% of subjects recovered normosmia over 7 to 8 weeks after symptoms onset. Another study in Italy [13] showed that 67% of patients had olfactory dysfunction at symptoms onset, and it was still present in 56% after 20 days. These studies concluded that a high proportion of patients recovered olfactory function in a short time interval; however, many patients still had a detectable olfactory dysfunction. In our study, 19% of subjects presented olfactory dysfunction after a median follow-up of 4 months. It must be recognized that three of the five hyposmic patients scored just one error above their threshold for age, indicating a very mild dysfunction. Because we could not test subjects of the recovered group in the acute phase of the disease, we can only report subjective information about their initial olfactory dysfunction. However, it should be highlighted that the proportion of recovered subjects who reported olfactory dysfunction during acute illness (58%) is very similar to the proportion of hyposmic hospitalized patients as measured with olfactory testing (64%). Since the congruence between subjective symptoms and performance at olfactory testing seems to be high in the “recovered” group, the proportion of these subjects reporting olfactory dysfunction during the acute phase could be considered as reliable. One study showed low agreement between self-reported olfactory function and olfactory testing [18]. However, patients were tested a mean of 18 days after the onset of olfactory dysfunction and psychophysical testing was carried out with a suprathreshold identification test, therefore some patients reporting subjective olfactory dysfunction might have already recovered or might have had an isolated olfactory threshold disorder. In our study, in order to accurately collect each participant’s olfactory history and nasal symptoms during acute illness and at the time of olfactory testing, we used a structured questionnaire and the total nasal symptom score.

Overall, our findings extend current knowledge on COVID-19 olfactory dysfunction by providing evidence that olfactory dysfunction can still be present in a non-negligible fraction of patients 4 months after symptoms onset and may therefore be regarded as a long-term sequela of the disease. Furthermore, we documented a highly variable subjective recovery time, from 9 days to more than 3 months. Subjective patient history and psychophysical test performed in this study suggest that some patients may need even longer time and their possibility of full recovery is currently unknown. Indeed, the pathophysiology of COVID-19-associated olfactory dysfunction is still being investigated. Evidence from autoptic series has shown a high degree of inflammation, astrogliosis, and microgliosis in the olfactory bulb of deceased COVID-19 patients [19, 20], while MRI studies have shown an early, reversible, obstruction of the olfactory cleft as a possible short-term cause of olfactory dysfunction [21, 22]. One study has pointed out that olfactory dysfunction may derive from a viral insult to olfactory neurons supporting cells [23], although an autopsy-based investigation found viral RNA in the olfactory mucosa just beneath the olfactory cleft as well as in neuroanatomical structures receiving olfactory projections, i.e., the olfactory bulb [24]. Finally, a recent study has shown significant histological recovery of olfactory structures in a COVID-19 animal model about 3 weeks after infection [25].

Several limitations must be acknowledged. This study was cross-sectional, therefore we were unable to report prospective results on patients who were “objectively” hyposmic during the acute phase of the disease. However, careful histories of olfactory dysfunction were collected in both groups included in our study. Psychophysical olfactory testing was performed with a suprathreshold identification test, therefore we were unable to test other olfactory characteristics, i.e., olfactory threshold. Although we collected the total nasal symptom score at the time of olfactory testing, rhinoscopy was not performed in this study, therefore we cannot rule out silent rhinosinusal diseases in some subjects as a cause for olfactory dysfunction; especially in the recovered group, however, all subjects who experienced olfactory loss reported a sudden and profound dysfunction, thus rendering unlikely etiological hypotheses other than SARS-CoV-2 insult. The two cohorts included different profiles of COVID-19 patients: hospitalized patients represent a moderate-to-severe spectrum of the disease; recovered subjects are more heterogenous, including five asymptomatic subjects and two subjects who had been hospitalized during the acute phase of the disease for respiratory distress; overall, recovered subjects represent a population with a milder COVID-19 phenotype. Furthermore, hospitalized patients were tested in an acute setting, which might have increased the intrinsic difficulty of the test; however, inclusion and exclusion criteria were designed in order to exclude patients with a history of neurological disease (especially dementia and parkinsonism) and/or those unable to breathe without supplementary oxygen for at least 30 minutes. All olfactory tests were conducted by physicians with specific training for IOIT administration who verified patients’ fitness to adequately undergo olfactory testing. Although subjects in the recovered group were randomly selected among employees that had a positive antibody COVID-19 assay, it is possible that subjects with previous or current olfactory dysfunctions were more willing to participate. Therefore, the proportion of hyposmic subjects in the recovered group, coupled with the relatively small sample size, may be an overestimation and must be interpreted with caution. Previous population studies have reported a prevalence of olfactory dysfunction in about 20% of the general population when olfactory psychophysical testing was carried out with suprathreshold tests [26, 27]. In a series from two of the authors (C.M., S.S.) in which more than 1000 Italian healthy volunteers were screened with the same psychophysical olfactory test, around 12% had an abnormal test score and was thus hyposmic (unpublished data). Therefore, the frequency of hyposmia in the recovered group, which includes many subjects with previous COVID-19-related olfactory symptoms, seems to be slightly higher to that of the general population. Finally, evidence of prior SARS-CoV-2 infection was detected through either one of two commercial antibody assays with specificities of 98.5% and 99.8%. Although possible, it is unlikely to have included false positive subjects in the recovered group.

## Conclusion

In this study we used a standardized, 33-item psychophysical olfactory identification test tailored for the Italian population in order to investigate COVID-19-related olfactory dysfunction in 51 subjects among acute hospitalized patients and recovered subjects. Moderate-to-severely ill COVID-19 patients showed low awareness and a high frequency of olfactory dysfunction (64% of patients). Interestingly, the proportion of recovered subjects who reported olfactory dysfunction during acute illness (58%) was very similar to the proportion of hyposmic hospitalized patients as measured with olfactory testing (64%). Recovered subjects showed good self-awareness of olfactory dysfunction and variable time, from days to months, to complete recovery; four of 15 (27%) recovered subjects who reported olfactory loss during acute COVID-19 still had an abnormal test score on psychophysical testing, indicating that olfactory dysfunction may be among the long-term sequelae of COVID-19. Large prospective studies based on threshold, discrimination, and identification psychophysical olfactory tests are needed to elucidate the clinical characteristics and trajectory of olfactory dysfunction in COVID-19.

## Supplementary Information

ESM 1 (DOCX 16 kb).

ESM 2 (DOCX 30 kb).

## Data Availability

Data used in the preparation of this manuscript is available upon request.

## Abbreviations

COVID-19: Coronavirus disease 2019
NAAT: Nucleic acid amplification test
SARS-CoV-2: Severe acute respiratory syndrome coronavirus 2
TNSS: Total nasal symptom score
