# Supplementary material for: Testing olfactory dysfunction in acute and recovered COVID-19 patients: a single center study in Italy
Source: Neurol Sci. 2021 Mar 26;42(6):2183–9. doi: 10.1007/s10072-021-05200-7 (PMC7994059; doi:10.1007/s10072-021-05200-7)
Supplement: Supplementary file 1 — (DOCX 16 kb). [file 10072_2021_5200_MOESM1_ESM.docx]

**SUPPLEMENTARY MATERIAL**

1. **Structured interview administered to patients at the time of psychophysical olfactory testing**

Prior to olfactory testing, the patient was required to answer the following questions in a yes/no fashion. All questions were assessed whether the patients reported normal or impaired smell and/or taste.

Questions regarding altered sense of smell: “Compared to the time prior the onset of COVID-19 symptoms:

- Do you feel like you have experienced a loss or alteration in the sense of smell?
- Do you think that you are able to normally feel the smell of food (e.g. coffee, fish, onion)?
- Do you think that you are able to normally feel unpleasant odors (e.g. toilet smells)?”

Questions regarding altered sense of taste: “Compared to the time prior the onset of symptoms of COVID-19 symptoms:

- Do you feel like you have experienced a loss or alteration in the sense of taste?
- Do you think that you are able to normally feel the taste of food (e.g. coffee, salt, spiced foods)?”

1. **Total Nasal Symptoms Score (Downie et al., 2004*)**

In this questionnaire nasal obstruction, itching/sneezing and secretion/runny nose are scored separately on a severity scale between 0 and 3, as follows:

- 0 = no symptom;
- 1 = mild – awareness but not troubled;
- 2 = moderate – troublesome but not interfering with normal daily activities or sleep;
- 3 = severe – interfering with normal daily activities or sleep.

Each score is summed to give a total score between 0 and 9.

*Downie, S.R., Andersson, M., Rimmer, J., Leuppi, J.D., Xuan, W., Akerlund, A., Peat, J.K. and Salome, C.M. (2004), Symptoms of persistent allergic rhinitis during a full calendar year in house dust mite‐sensitive subjects. Allergy, 59: 406-414. doi:10.1111/j.1398-9995.2003.00420.x

1. **Full list of odors of the Italian Olfactory Identification Test (IOIT)**

Clove, rose, lavender, banana, fir/pine tree, mushroom, talc, mint, coconut, strawberry candy, apple, cheese, watermelon, fresh-cut grass, violet flowers, sage, licorice, laundry soap, wood-like smell, coffee, chocolate powder, oregano, basil, rosemary, garlic, lemon, peach, incense, orange, anise-sambuca, pineapple juice, eucalyptus candy, unpleasant odor.

**SUPPLEMENTARY TABLES**

**Supplementary Table 1.** Results of statistical tests investigating the relationship between IOIT variables and clinical variables in acutely ill COVID-19 patients.

| **Variables tested** | **Statistical test** | **Test statistic** | **p-value** |
| --- | --- | --- | --- |
| Errors at IOIT, age | Spearman rank correlation | ρ = 0.368 | 0.071 |
| Errors at IOIT, disease duration | Spearman rank correlation | ρ = -0.34 | 0.878 |
| Presence of olfactory disturbance at IOIT, need for mechanical ventilation^a^ | Fisher’s exact test | χ^2^ = 0.322 | 0.688 |
| Number of errors at IOIT, need for mechanical ventilation^a^ | Binary logistic regression | OR = 0.933 (95% CI: 0.823 – 1.057) | 0.274 |

Abbreviations: CI: confidence interval; OR: odds ratio.

^a^dependent binary variable: no need for ventilation and need for ventilation (either invasive or non-invasive).
